# Through restful waters and deep commotion: A study on burnout and health impairment of Italian seafarers from the JD-R model perspective.

**DOI:** 10.12688/f1000research.159198.2

**Published:** 2025-02-20

**Authors:** Francesco Buscema, Lorenzo Cena, Clarissa Cricenti, Margherita Zito, Lara Bertola, Lara Colombo

**Affiliations:** 1 Psychology, University of Turin Department of Psychology, Turin, Piedmont, Italy; 2 Department of Psychology, University of Rome La Sapienza, Rome, Italy; 3 Department of Business, Law, Economics and Consumption, IULM, Milan, Lombardy, Italy; 4 Department of Management and Organization, Rennes School of Business, Rennes, Brittany, France

**Keywords:** Burnout, Seafarers, Health Impairment, JD-R Model, Quality of Sleep

## Abstract

**Background:**

The work experience of seafarers differs significantly from other land-based occupations due to several factors, particularly remoteness and the restricted work environment. This study seeks to examine the impact of burnout and health impairment in the maritime industry, using the Job Demand-Resources theory as a framework.

**Methods:**

To investigate these phenomena, an online questionnaire was sent to 629 Italian seafarers and we conducted analysis on a valid sample of 239 respondents (94.6% men, Mage = 39.44, SD = 12.8). We tested a mediated Structural Equation Model (SEM) aimed at predicting negative health outcomes.

**Results:**

The results show that burnout plays a mediating role between job demands (such as workload and cognitive strain) and health impairment (such as sleep quality and physical well-being) (Total Indirect Effect = 0.443, *p* < .0001) as well as between job resources (such as social support and transformational leadership) and health impairment (Total Indirect Effect = -0.249, *p* < .0001). Furthermore, the findings highlight the direct influence of occupational resources on seafarers’ health.

**Conclusions:**

The discussion highlights the urgent need for more research in the field of organisational psychology in the maritime industry and the discrepancies between these findings, which are consistent with the existing maritime literature, and other studies that do not include seafarers in their sample groups.

Working life at sea is very different from other jobs on land for many reasons, such as isolation and confined working environments. Stressors related to the characteristics of the work environment, such as noise, heat, cold, ship motion, jet lag, can affect quality of life ( Oldenburg et al., 2010), but also living and working in the same confined environment with the same crew members for a long period of time is considered a challenging task that affects mental health ( Sandal et al., 2006). In addition, characteristics of work in the maritime industry, such as the schedule of activities on board, are associated with difficulties at work, workload and circadian disturbance ( Pauksztat, 2017). These factors not only affect the safety of crew members and the quality of relationships on board, but also increase fatigue and health impairment ( Bakker et al., 2023; Pauksztat et al., 2022).

Currently, there are few articles analysing the quality of life and mental health of Italian seafarers ( Buscema et al., 2023; Carotenuto et al., 2012; Tedesco et al., 2018). There is a need to conduct more studies to increase the knowledge about the quality of life of Italian seafarers on board. Stressors, such as prolonged isolation, demanding physical tasks and the psychological strain of working in a confined environment, are exacerbated by the limited availability of resources, such as social support and effective leadership, which are critical to mitigating the negative effects of these demands. Considering this, the JD-R model is particularly relevant in this context as it assumes that the balance between job demands and resources has a direct impact on employee well-being and stress levels. The aim of this study is to investigate the main factors affecting burnout and health impairment among Italian seafarers, using the theoretical framework of the Job Demands-Resources model ( Bakker et al., 2023). Following the recent study by Wan et al. (2023) on Chinese seafarers, the aim of this paper is to understand the relationship between job demands, job resources, burnout and health impairments. Compared to the study by Wan et al. (2023), which identifies the factors related to mental health and work ability in the maritime industry, focusing on the role of the environment, we focus on outcomes related to health impairments such as bad quality of sleep and physical health. By applying this model to the maritime sector, we can better understand how specific job resources can alleviate the psychological and physical stress of seafarers and thus promote a healthier working environment.

## Literature review

### Seafarers’ mental health

Seafaring is a demanding profession both mentally and physically. The physical risks associated with maritime occupations, such as musculoskeletal problems, have been widely studied in the literature ( Hansen et al., 2008; Remmen et al., 2023; van de Wijdeven et al., 2023), while psychosocial risk factors and dimensions of mental health have only been addressed in recent years, with a focus on specific issues such as burnout ( Chung et al., 2017; Oldenburg et al., 2010, 2014; Wan et al., 2023), fatigue ( Abila & Acejo, 2021; Oldenburg & Jensen, 2019) and sleep quality ( Hystad & Eid, 2016; Lützhöft et al., 2011). Nevertheless, the number of studies dealing with these topics is still small and there are even fewer studies in the Italian context.

Seafarers may develop psychological or post-traumatic symptoms after experiencing a pirate attack during their working hours ( Abila & Tang, 2014). A recent report on seafarers’ mental health shows that the prevalence of depression and anxiety affected 28% of the 1262 seafarers surveyed in 2019 ( Lefkowitz & Slade, 2019). Some studies show that the factor that has the greatest impact on seafarers’ quality of life on board and mental health is isolation from family ( Buscema et al., 2023; Lefkowitz & Slade, 2019).

During the COVID-19 pandemic, the maritime sector played a key role in the survival of the global economy. However, seafarers faced problems that affected their mental health, such as the inability to change shifts, shortages of supplies on board, increased workloads and overdue services ( Baygi et al., 2021; Brooks & Greenberg, 2022; Pauksztat et al., 2022). Our study will aim to deepen the knowledge of psychosocial risk factors and possible factors that mediate the well-being of seafarers.

### JD-R Model

Job Demands-Resources (JD-R) theory is a model of occupational psychology that assumes that the balance between job demands and individual and contextual resources influences work stress and well-being ( Bakker et al., 2023). This theory is able to overcome the limitations of the most common theories of work stress, such as the two-factor theory ( Herzberg, 1966), the job characteristics theory ( Hackman & Oldham, 1976), the job demand-control model ( Karasek, 1979), the effort-reward-imbalance model ( Siegrist, 1996) and conservation of resources theory ( Hobfoll et al., 2018). JD-R theory is general enough to be applied to all jobs and fits the project design as it can explain the job characteristics of seafarers through the categories of job demands and job resources. In addition, JD-R examines the process of health impairment and motivation, personal resources such as self-efficacy, resilience and humour at work, and the role of exhaustion in job performance ( Bakker et al., 2023).

Few studies have examined the role of job resources on shipboard mental health using the JD-R model, suggesting that social support (from peers and external support) and the opportunity to go ashore for holidays, as well as access to communication and entertainment, may reduce mental health problems in seafarers ( Pauksztat et al., 2022; Tang et al., 2022). Another study examines the role of JD-R among cruise ship crew and shows that the negative effects of job demands on work engagement are mitigated by workers’ individual strategies, such as recovery or work-related effort ( Radic et al., 2020). A recent study investigates the moderating role of a fun environment onboard between job demands and turnover intentions. Specifically, the condition of low job demands and high fun environment, turnover intentions are significantly lower than in the low fun environment condition ( Gu et al., 2020). To the best of our knowledge, this study is the first to test the JD-R model on Italian seafarers and thus enriches the extant literature.

The activities of seafarers on board could be associated with a hierarchical environment in which the roles of all crew members are not interchangeable. Leaders or supervisors play a crucial role in steering not only the ship but also the crew members. For this reason, leadership styles such as transformational leadership could be very effective on board to prevent safety issues and motivate seafarers ( Sandhåland et al., 2017). Transformational leadership can be summarised as a leadership model consisting of four factors: idealised influence, inspirational motivation, intellectual stimulation and individualised consideration ( Bass & Riggio, 2006).

## Research hypotheses and model construction

### Work and cognitive load

According to the JD-R model ( Bakker et al., 2023), work and cognitive load are considered predictors of stress, burnout and health impairment in the workplace. A recent study confirms the role of high workload as a predictor of burnout in a large sample of Chinese seafarers ( Wan et al., 2023). In addition, the role of human factors, such as cognitive load, is considered to be one of the most important dimensions influencing workplace safety, especially in the maritime environment ( Seyfzadehdarabad et al., 2023). Fatigue and stress could be considered as consequences of high cognitive load over long periods of time in seafarers ( Žagar et al., 2020). Hypothesis 1: Work and cognitive load (job demands) are positively related to health impairments through the mediation of job burnout.

### Social support and transformational leadership

Living in an isolated environment for so long means that every seaman has to deal with every crew member. The hierarchical way in which seafarers are managed on board is a crucial issue for the quality of life of all seafarers. For this reason, leadership and social support could be considered as such resources according to the JD-R model ( Bakker et al., 2023). Transformational and authentic leadership can be considered a resource that promotes the development of psychological capital and creativity according to recent studies ( Rego et al., 2012; Yuen et al., 2020). By embodying social support and transformational leadership, crew members should be able to create a positive work environment that acts as a resource against burnout and health impairment ( Lucas et al., 2021; Sampson & Zhao, 2003; Wan et al., 2023). Hypothesis 2: Social support and transformational leadership (job resources) are negatively related to health impairments through the mediation of job burnout.

### Hypothetical model

**Figure 1.**
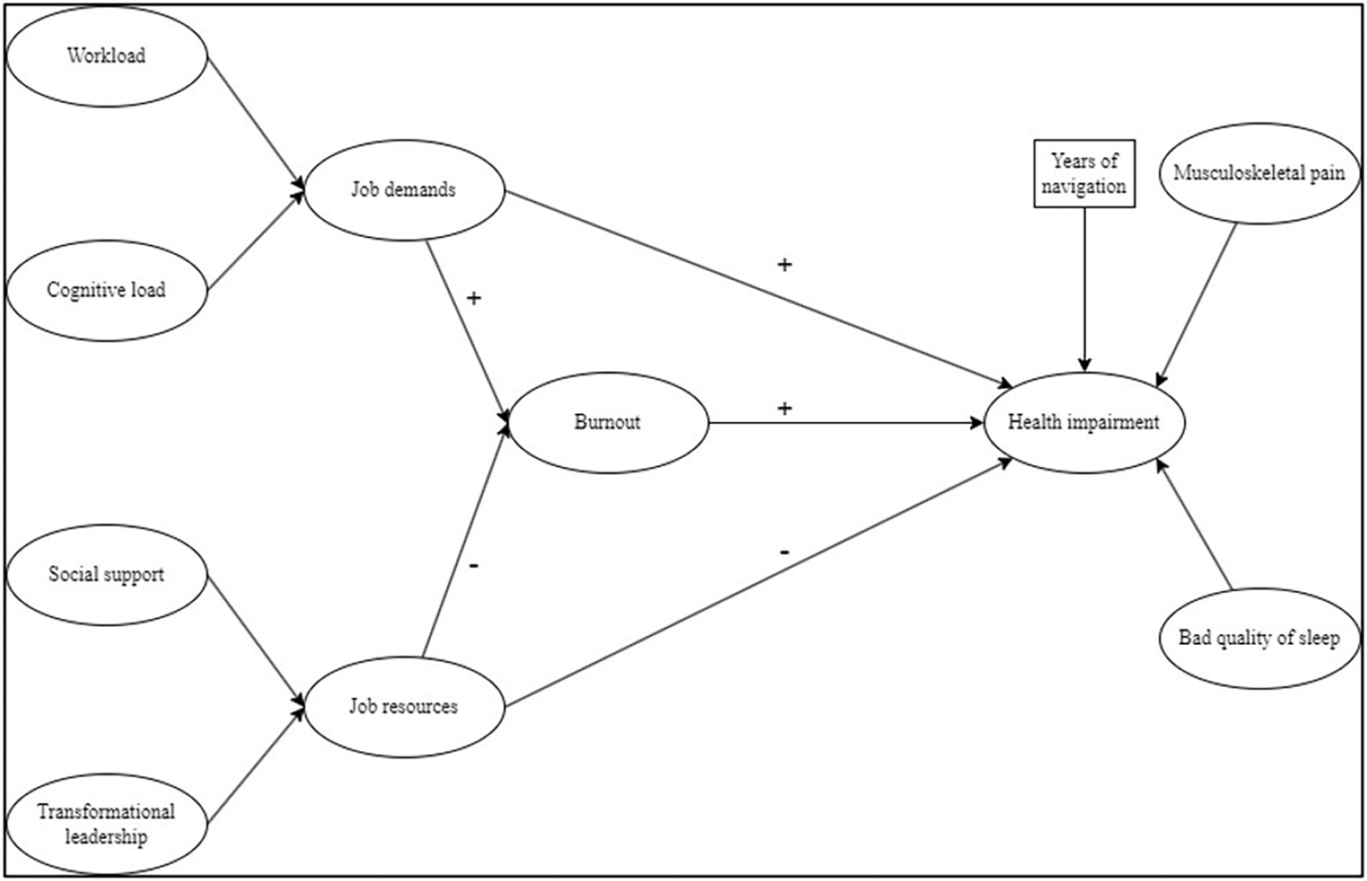
Hypothetical model based on JD-R model. Note. Ovals represent latent variables; square represents manifest variable.

The model aims to analyse the mediating role of burnout between the JD-R dimensions and health impairments, using years of navigation as a control variable, as shown in Figure 1.

## Methods

### Data collection and respondents

An online survey was conducted from February to May 2023 using the Qualtrics XM platform. The questionnaire used the JD-R model ( Bakker et al., 2023) to investigate psychosocial risks on board. Respondents were recruited through snowball sampling and participation was voluntary. Through a dedicated website and thanks to the support of the unions in disseminating the questionnaire, 629 seafarers from all departments and ranks responded to the questionnaire. Informed written consent was obtained from all participants prior to data collection. After excluding cases with missing values and workers employed in the catering department, the total valid sample for the present analysis was 239 seafarers. The study protocol was approved by the Ethics Committee [Prot. n. 0513027 del 03/10/2022 - UOR: SI000045—Classif. III/11]. Data analysis was conducted using IBM SPSS version 28.0.1.1 and MPlus version 8.

The gender distribution shows that 5.4% of the sample is female, reflecting the gender gap in the maritime environment ( Baltic and International Maritime Council & International Chamber of Shipping, 2021). The average age was 39.44 years (SD = 12.81 years). Concerning the marital status of our sample, 47.7% were married, 25.1% were engaged, 20.5% were single, while the rest were divorced or widowed.

All ranks of deck and engine department were included in the study: 64.4% of seafarers were employed in the deck department. 18.8% of the seafarers were Captains, 30.1% were deck officers, 6.3% were deck cadets, 8.8% were deck ratings, 9.2% were Chief engineers, 14.6% were engine officers, 10.9% were engine ratings. 51.9% of the participants had more than ten years’ experience in shipping. Furthermore, 64.9% of the sample responded to the survey while on board.

The means and standard deviations of all the constructs studied are presented in Table 1, while Table 2 presents the correlations between all the constructs studied. We tested our hypotheses using a structural equation model (SEM) with MPlus version 8. We implemented bootstrap method to create 2000 bootstrap samples ( Preacher & Hayes, 2008). Estimates of the indirect effect composed of the products of alpha paths (i.e., from job demands and job resources to burnout) and the beta path (i.e., from burnout to health impairment) were estimated with the associated 95% confidence interval (CI). If the CI does not include zero, then we consider the indirect effect to be statistically significant ( *p* < .05).

**Table 1.** Means, standard deviations of the constructs studied.

Constructs	Mean	S.D.
Workload	4.26	1.23
Cognitive load	4.29	0.81
Social support	2.89	1.07
Transformational leadership	3.01	1.10
Burnout	3.09	1.02
Musculoskeletal pain	2.64	0.95
Quality of sleep	3.81	1.15

*Note*: This table shows means and standard deviation of the constructs studied in the questionnaire on a sample of 239 seafarers.

**Table 2.** Correlation between model constructs.

	*N*	*M*	*SD*	1	2	3	4	5	6	7
1.Workload	239	4.20	1.21	--						
2.Cognitive load	239	4.21	0.86	.64 **	--					
3.Social support	239	2.88	1.08	-.24 **	-.10	--				
4.Transformational leadership	239	3.02	1.09	-.13	-.04	.47 **	--			
5.Burnout	239	3.05	0.99	.46 **	.30 **	-.45 **	-.27 **	--		
6.Musculoskeletal pain	239	2.68	0.98	.33 **	.26 **	-.29 **	-.12	.47 **	--	
7.Quality of sleep	239	3.72	1.15	.50 **	.37 **	-.46 **	-.35 **	.68 **	.57 **	--

** *p* value < 0.01.

* *p* value < 0.05.

### Questionnaire and measures

Based on the studies related to the JD-R model, we selected as variables for job demands: Workload and Cognitive Load. Job resource variables were social support and transformational leadership, while the outcome variables were burnout and health impairment. Years of navigation were used as control variables.

**Job demands**

The Italian adaptation of the Psychological Workload and Physical Workload subscales of the Job Content Questionnaire (JCQ), which is one of the most common instrument for assessing workload, were used to examine work and cognitive load ( Baldasseroni et al., 2001; Karasek et al., 1998). 6 items were used to examine workload (e.g., “I have too much work to do”) (Cronbach’s α = 0.882), while 4 items examined cognitive load (e.g., “My work requires my constant attention”) (Cronbach’s α = 0.831). All items on workload are rated on a 6-point scale from *completely disagree* (1) to *completely agree* (6), while the items on cognitive load are rated on a 5-point scale from *completely disagree* (1) to *completely agree* (5).

**Job resources**

Based on a previous study of the maritime sector, the following scales were selected to examine social support and transformational leadership ( Yuen et al., 2020).

5 Items from the Multidimensional Perceived Social Support Scale (MSPSS) ( Dambi et al., 2018; Zimet et al., 1988) were used to assess on-board social support (e.g., “I receive emotional help and support I need from my teammates”). The original MSPSS includes not only perceived social support from colleagues, but also from family and friends. Following Yuen et al. (2020), it was decided to use only social support from colleagues. The items are measured on a 5-point rating scale from strongly disagree (1) to strongly agree (5) (Cronbach’s α = 0.916).

5 items from the Multifactor Leadership Questionnaire ( Schuckert et al., 2018) were used to explore transformational leadership on board (e.g., “My superior spends time teaching and coaching”). These items relate to: attributed idealised influence, behavioural idealised influence, inspirational motivation, intellectual stimulation and individualised consideration. The scale is measured on a 5-point rating scale from not at all (1) to always (5) (Cronbach’s α = 0.890).

**Outcomes**

We used the Maslach Burnout Inventory ( Maslach & Leiter, 2016) to measure burnout on board, using 6 items as conducted by Yuen et al. (2020). Burnout is composed of emotional exhaustion (e.g., “I feel emotionally drained from work”), depersonalisation (e.g., “I worry that this job is hardening me emotionally”), and reduced achievement (e.g., “I have accomplished many worthwhile things in this job”). The items are measured on a 5-point rating scale from never (1) to always (5). To increase the reliability of burnout, the item “I have accomplished many worthwhile things in this job” was removed from the analysis Cronbach’s α increases from 0.726 to 0.780.

According to the JD-R theory ( Bakker et al., 2023) and following job design of Wan et al. (2023), health impairment was measured by two different indicators: musculoskeletal pain and bad quality of sleep. Musculoskeletal pain was measured using 8 items from the psycho-physical symptoms indicator (e.g., “How often did you suffer the following symptoms: Muscle and joint pain”) by Avallone and Paplomatas (2004) (Cronbach’s α = 0.867). Items are measured on a 5-point rating scale from never (1) to always (5). The Italian adaptation of the Mini Sleep Questionnaire ( Natale et al., 2014) was used to assess the quality of sleep on board. This scale consists of 10 items (e.g., “I have had problems sleeping”) measured on a 7-point rating scale from never (1) to always (7) (Cronbach’s α = 0.894).

## Results

The model showed good fit indices CFI = 0.909, TLI = 0.903, χ ^2^ (883, N =239) = 1466.436, *p* < .0001, SRMR = 0.071, RMSEA = 0.053, Confidence Interval (C.I.) = .048 - .057, according to Hayduk et al. (2007) and Kline (2005). CFI and TLI have values that are above the cut-off (0.90), while SRMR and RMSEA are acceptable as the values are below the cut-off (.08 and .05 respectively), indicating that the model has a good fit ( Hu & Bentler, 1999).

In our measurement model, we used our items to model the constructs as latent variables using CFA. All factor loadings were significant (less than *p* < .001) and are listed in Table 3.

**Table 3.** Loading of items on factors studied.

Construct	Item/Factor	Estimate	S.E.	*p*-value
Workload	Q7	0.550	0.058	<0.001
	Q8	0.659	0.052	<0.001
	Q9	0.874	0.027	<0.001
	Q10	0.854	0.031	<0.001
	Q11	0.688	0.048	<0.001
	Q12	0.762	0.035	<0.001
Cognitive load	Q13	0.837	0.034	<0.001
	Q14	0.644	0.062	<0.001
	Q15	0.664	0.065	<0.001
	Q16	0.686	0.062	<0.001
Social support	Q17	0.873	0.024	<0.001
	Q18	0.913	0.015	<0.001
	Q19	0.883	0.023	<0.001
	Q20	0.740	0.037	<0.001
	Q21	0.666	0.045	<0.001
Transformational leadership	Q22	0.756	0.045	<0.001
	Q23	0.630	0.059	<0.001
	Q24	0.840	0.035	<0.001
	Q25	0.870	0.026	<0.001
	Q26	0.815	0.035	<0.001
Burnout	Q27	0.824	0.030	<0.001
	Q28	0.818	0.033	<0.001
	Q29	0.602	0.056	<0.001
	Q30	0.685	0.044	<0.001
	Q32R	0.241	0.072	<0.001
Musculoskeletal pain	Q33	0.412	0.062	<0.001
	Q34	0.619	0.048	<0.001
	Q35	0.871	0.024	<0.001
	Q36	0.761	0.039	<0.001
	Q37	0.712	0.039	<0.001
	Q38	0.686	0.046	<0.001
	Q39	0.726	0.044	<0.001
	Q40	0.447	0.059	<0.001
Bad quality of sleep	Q41	0.649	0.042	<0.001
	Q42	0.665	0.044	<0.001
	Q43	0.421	0.050	<0.001
	Q44	0.713	0.034	<0.001
	Q45	0.785	0.028	<0.001
	Q46	0.287	0.067	<0.001
	Q47	0.643	0.041	<0.001
	Q48	0.757	0.031	<0.001
	Q49	0.892	0.019	<0.001
	Q50	0.837	0.024	<0.001
Job demands	Workload	0.943	0.054	<0.001
	Cognitive load	0.824	0.051	<0.001
Job resources	Social support	0.859	0.084	<0.001
	Transformational leadership	0.628	0.066	<0.001
Health impairment	Musculoskeletal pain	0.632	0.059	<0.001
	Bad quality of sleep	0.949	0.037	<0.001

*Note.* This table describe the loading effect of factors studied in the questionnaire. First column shows the name of the constructs while second show the name of the items and of the factors. The last three columns show the loading effect, the standard deviation, and P-Value.

The results of the path coefficients of the model were mostly significant at *p* < 0.001, with the exception of the direct effect of job demands on health impairment and the effect of the control variable (years of navigation) on health impairment ( Table 4). Confirming the theoretical model of JD-R, job demands (work and cognitive load) were positively associated with burnout (estimate = 0.622, *p* < 0.001), while job resources (social support and transformational leadership) were negatively associated with burnout (estimate = - 0.350, *p* < 0.001). Moreover, burnout was strongly and positively associated with health impairment (estimate = 0.713, *p* < 0.001).

**Table 4.** The structural model.

Model path	Estimate	S.E.	*p*-value
Work and Cognitive load on Burnout	0.622	0.068	<0.001
Work and Cognitive load on Health impairment	0.040	0.102	0.693
Social support and Transformational leadership on Burnout	-0.350	0.068	<0.001
Social support and Transformational leadership on Health impairment	-0.217	0.091	0.018
Burnout on Health impairment	0.713	0.134	<0.001
Year of navigation on Health impairment ( *control variable*)	-0.079	0.056	0.154

*Note.* This table describe the loading effect, standard deviation, and p-value for the model path. Last row concern the control variable of the model.

**Figure 2.**
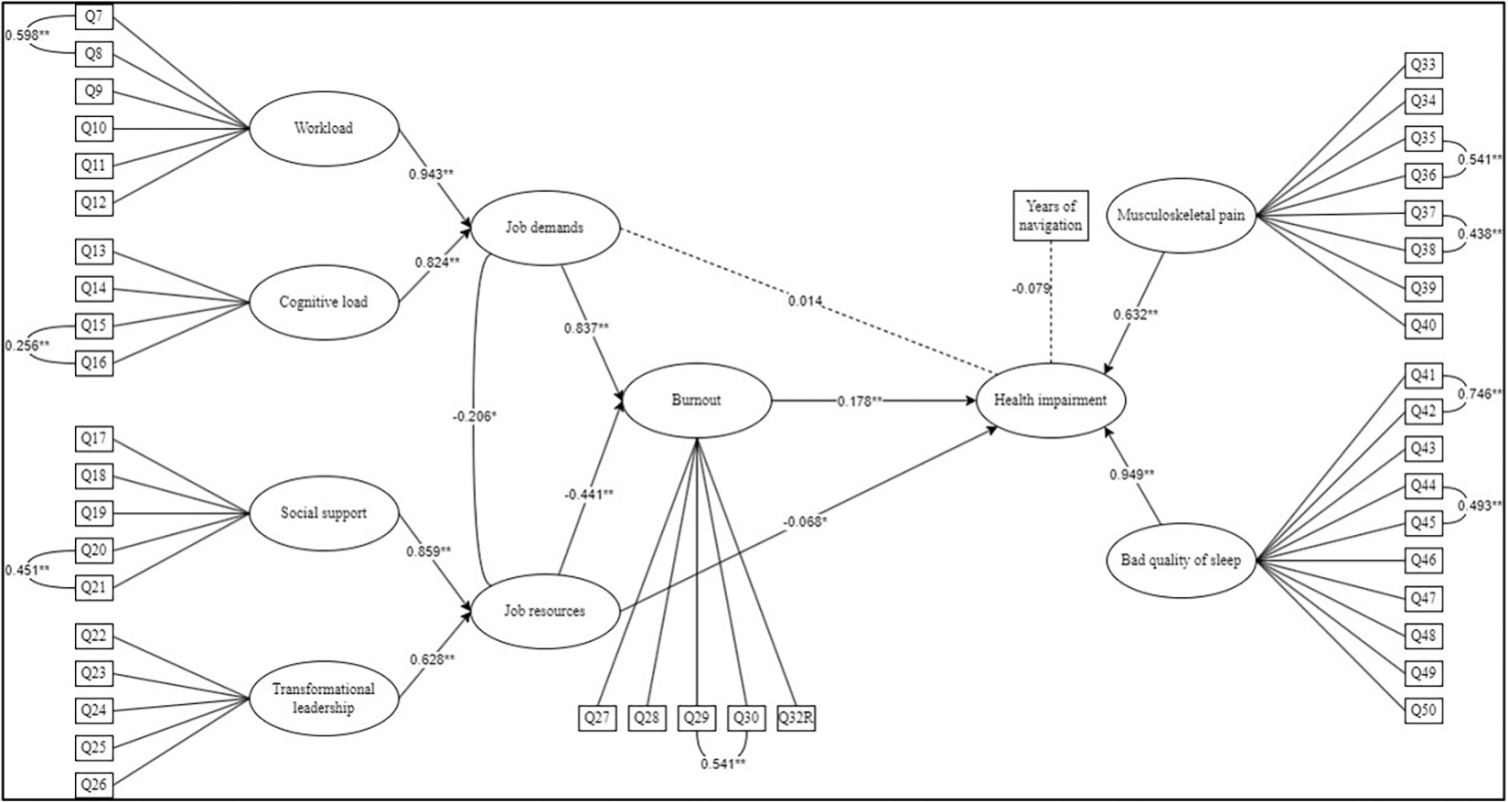
The estimated JD-R model. *Note.* Dotted lines represent not significant relationships. Ovals represent latent variables; square represents manifest variable. Significance is represented as follow: * *p* < 0.05; ** *p* < 0.001.

We analysed the standardised total and specific indirect effects. The results ( Figure 2) show that the C.I. for the two indirect effects do not include zero, confirming both hypotheses of mediation at a significant level ( *p* < .05). First, burnout fully mediates the relationship between job demands and health impairment (Standardised Total Indirect Effect = 0.443, *p* < .0001, C.I. = 0.259 to 0.682), as the specific direct effect of job demands on health impairment is not significant ( *p* = .69). Second, burnout partially mediates the relationship between job resources and health impairment (Standardised Total Indirect Effect = -0.249, *p* < .0001, C.I. = -0.398 to -0.143), because the direct effect of job resources on health impairment is still significant when the indirect effect of mediation is taken into account (estimate = 0.217, *p* = .018).

Our model explains 77.4% of the variance in health impairment. We confirmed the full mediation of hypothesis 1 and partially confirmed hypothesis 2 because the mediation is not full.

## Discussion

The aim of our study was to understand the relationship between burnout, JD-R variables and health impairment in the maritime industry. Our findings, which follow recent studies on these topics ( Bakker et al., 2023; Yang & Hayes, 2020), confirm that burnout plays a crucial role in predicting health impairments in the seafarer population as well. We have gained valuable insights into the dynamics at play in this particular work environment.

The model fits the data well and explains much of the variance in our independent variables. Both hypotheses were fully or partially confirmed.

According to the JD-R theory, job demands are composed of two dimensions of workload: physical and cognitive. In addition, job demands were expected to have a direct effect on health impairment, as severe job demands have been found to lead to health impairment in the literature ( Bakker et al., 2023). According to the process outlined by JD-R theory, frequency and severity of job demands lead to an increase in effort, which depletes workers’ physical and cognitive resources, leading to exhaustion and health impairment. Our findings deepen our knowledge of this process, as in our study severe job demands only lead to health impairments through the mediation of burnout.

The partial mediation of burnout that we found between job resources and health impairments is also related to recent studies in the maritime industry that have found a direct relationship between job resources and health impairments ( Lucas et al., 2021; Sampson & Zhao, 2003; Wan et al., 2023). This finding suggests that in the maritime industry, job resources such as social support and transformational leadership have a direct influence on the prevention of health impairments, while in other occupations this relationship was hardly observed ( Alarcon, 2011; Mayerl et al., 2016).

Our study has shown that burnout is a crucial variable in mediating between job demands and resources and negative health outcomes. More specifically, this study has identified two different ways in which job resources predict health impairment. First, the direct effect of resources on reducing health impairment, and second, a mediated effect via burnout. For example, a scenario where a group of seafarers with a transformative leadership style on board and a supportive work environment between all crew members could limit the risk of an increase in health impairment both directly and indirectly by reducing negative psychological experiences such as burnout. In this way, the support and positive atmosphere created by leadership and crew members act as a buffer against the negative health consequences of the demanding work environment, leading to improved overall seafarer well-being. Identifying and addressing these factors can be critical to developing effective interventions and strategies to promote better occupational health in the maritime industry. It is crucial to design and implement training programmes that focus on these aspects which can empower ship masters to create a supportive environment that encourages open communication, recognition of individual contributions and collective problem solving. For example, leaders who practise transformational leadership can actively engage crew members in decision-making processes, thereby increasing their sense of ownership and reducing feelings of helplessness that often accompany high job demands ( Sætrevik & Hystad, 2017; Sandhåland et al., 2017).

Furthermore, improving social support systems on board ships is crucial for coping with the particular challenges seafarers face, such as isolation and confined living conditions. Research shows that social support from peers contributes significantly to psychological well-being and can mitigate the negative effects of job demands ( Brooks & Greenberg, 2022). Establishing structured peer support programmes where crew members can share their experiences and coping strategies can be helpful in promoting resilience. In addition, the integration of regular team-building activities can strengthen interpersonal relationships and thus improve the overall support network available to crew members.

Finally, it is important to note that the maritime industry has unique stressors that differ from those of the general employee population. For example, studies have shown that seafarers are more likely to suffer from mental health problems such as depression and anxiety, compared to land-based workers ( Makara-Studzińska et al., 2020). This discrepancy emphasises the need for tailored measures that take into account the specific demands of working at sea. By drawing parallels with findings from other high-stress professions such as healthcare ( Ghislieri et al., 2021) or professors ( Huynh et al., 2014), we can better understand the impact of burnout and health impairment among seafarers and advocate for industry-specific solutions ( Bakker et al., 2023).

## Conclusion

The findings highlight the need to further develop research on organisational psychology in the maritime industry. The results show the mediating role of burnout between job demands (workload, cognitive load) and resources (social support, transformational leadership) variables and health impairment variables (bad quality of sleep and health problems). Furthermore, the results highlight the direct role of job resources in preventing health impairment among seafarers. As we discussed in the previous section, our results show the incongruence between the results from a sample of seafarers and other samples from the general worker population.

There are some limitations to this study. All data were collected using an online self-report questionnaire on a voluntary basis, which means that participant-reported effects may be biased, especially for sensitive topics such as health and psychological well-being ( Roccato, 2003).

The burnout measure in the questionnaire was not developed for diagnostic purposes. In the questionnaire, burnout is measured by exploring symptoms, not in a clinical way ( Demerouti et al., 2021). Another limitation related to burnout is the possible overlap with the construct of depression. A recent meta-analysis shows that these two constructs are difficult to distinguish (correlation of r = 0.80), concluding that burnout problematically overlaps with depression ( Bianchi et al., 2021). Nevertheless, the ongoing exploration of burnout is crucial not only for distinguishing it from depression but also for developing effective strategies to support mental health specifically in high-stress occupations, such as seafarers ( Koutsimani et al., 2019).

Moreover, the sample is not representative of the total population of seafarers, as determining the exact number of seafarers is Italy is challenging due to the lack of a public register. A report from Confitarma (2019) suggested an estimate of 46,350 seafarers in Italy ( Duci et al., 2019). Furthermore, the particular nature of the maritime population does not allow for a multi-group analysis based on gender ( Baltic and International Maritime Council & International Chamber of Shipping, 2021). Finally, the data was collected using a cross-sectional design with a questionnaire that could not provide information on the causality of the variables.

Longitudinal studies should be conducted to investigate whether we can assume causality between the variables considered in this study. Other methods, such as qualitative interviews and diary studies, could deepen our knowledge of these issues.

Notwithstanding these limitations, the sample is one of the largest and most diverse in the Italian maritime sector to our knowledge, with an ongoing project to back it up that will lead to subsequent studies with qualitative and longitudinal approaches. In particular, this study presents new findings related to JD-R theory and contributes to the literature to increase knowledge related to maritime psychology. Based on the findings of this study, some practical conclusions can be drawn for the development of interventions that focus on strengthening on-board labour resources, such as social support and transformational leadership training. This study clearly shows that job demands such as work and cognitive load have a direct impact on seafarers’ psychological well-being (burnout), but are not directly related to their physical well-being. Interventions that improve relationships with colleagues and supervisors not only have an impact on reducing burnout, but also improve physical well-being by reducing musculoskeletal pain and improving sleep quality.

### Ethics

The study protocol was approved by the Ethics Committee [Prot. n. 0513027 del 03/10/2022 - UOR: SI000045—Classif. III/11].

### Consent to participate

Informed written consent was obtained from all participants prior to data collection. The procedure was approved by the Ethics Committee.

## Data Availability

Due to the data restriction policy aforementioned, readers and reviewers could contact the corresponding author (Francesco Buscema: francesco.buscema@unito.it) for dataset access. Supplementary material uploaded in Harvard Dataverse: https://doi.org/10.7910/DVN/RGH7ZO ( Buscema, 2024). This project contains the following extended data:
• Questionnaire: Survey_EN.pdf • Informed Consent: Informed Consent.pdf Data are available under the terms of the CC0 1.0 Universal license (CC0).
