# Supplementary material for: Through restful waters and deep commotion: A study on burnout and health impairment of Italian seafarers from the JD-R model perspective
Source: F1000Res. 2025 Feb 20;14:19. Originally published 2025 Jan 3. [Version 2] doi: 10.12688/f1000research.159198.2 (PMC11751612; doi:10.12688/f1000research.159198.2)
Supplement: Peer review history

# Reviewer report: Reviewer response for version 2

**DOI:** 10.5256/f1000research.177601.r367846

**Published:** 2025-02-28

**Authors:** Inga Bartuseviciene

**Affiliations:** 1 World Maritime University, Malmö, Sweden

In my view, the authors have properly addressed the comments and the paper is ready to be indexed.

Is the work clearly and accurately presented and does it cite the current literature?

Yes

If applicable, is the statistical analysis and its interpretation appropriate?

Yes

Are all the source data underlying the results available to ensure full reproducibility?

Partly

Is the study design appropriate and is the work technically sound?

Partly

Are the conclusions drawn adequately supported by the results?

Yes

Are sufficient details of methods and analysis provided to allow replication by others?

Yes

Reviewer Expertise:

Maritime Education and training, well-being and resilience

I confirm that I have read this submission and believe that I have an appropriate level of expertise to confirm that it is of an acceptable scientific standard.

# Reviewer report: Reviewer response for version 2

**DOI:** 10.5256/f1000research.177601.r367847

**Published:** 2025-02-27

**Authors:** Vairavan C

**Affiliations:** 1 AMET Deemed to be University, Chennai, Tamil Nadu, India

As per the recommendations all the points incorporated in the manuscript. No revisions.

Is the work clearly and accurately presented and does it cite the current literature?

Partly

If applicable, is the statistical analysis and its interpretation appropriate?

Yes

Are all the source data underlying the results available to ensure full reproducibility?

Yes

Is the study design appropriate and is the work technically sound?

Partly

Are the conclusions drawn adequately supported by the results?

Partly

Are sufficient details of methods and analysis provided to allow replication by others?

Yes

Reviewer Expertise:

Psychological well-being, burnout, and health impairment among maritime professionals.

I confirm that I have read this submission and believe that I have an appropriate level of expertise to confirm that it is of an acceptable scientific standard.

# Response

**Published:** 2025-02-25

**Authors:** Vairavan C

**Affiliations:** English, Academy of Maritime Education and Training, Kanathur, Tamil Nadu, India

Authors has revised and modified based on the comments.

# Reviewer report: Reviewer response for version 1

**DOI:** 10.5256/f1000research.174890.r358865

**Published:** 2025-01-21

**Authors:** Andrea Russo

**Affiliations:** 1 University of Split, Split, Croatia

The study is very interesting and makes a valuable contribution to the scientific literature.

 The number of respondents to whom the questionnaire was sent and the number of those who responded should be clearly stated in the sampling section. In the abstract, only the number of respondents who completed the questionnaire should be mentioned to avoid confusion for the reader.

 Regarding the statement: "There is no information on the real number of seafarers in Italy," a clearer explanation is needed. The concern is how this information is assessed, as seafarers are typically required to obtain certification, and there should be a sailor registry or similar official records.

Is the work clearly and accurately presented and does it cite the current literature?

Yes

If applicable, is the statistical analysis and its interpretation appropriate?

Yes

Are all the source data underlying the results available to ensure full reproducibility?

Yes

Is the study design appropriate and is the work technically sound?

Yes

Are the conclusions drawn adequately supported by the results?

Yes

Are sufficient details of methods and analysis provided to allow replication by others?

Yes

Reviewer Expertise:

Preservation of the mental health of seafarers

I confirm that I have read this submission and believe that I have an appropriate level of expertise to confirm that it is of an acceptable scientific standard.

# Response

**Published:** 2025-01-27

**Authors:** Francesco Buscema

**Affiliations:** Psychology, University of Turin Department of Psychology, Turin, Piedmont, Italy

Thanks for the valuable revision, we addressed the issues according to your suggestions as follows:

- Thank you for finding the mistake in the abstract, we clarified the total number of respondents by adding the following sentence in the abstract: To investigate these phenomena, an online questionnaire was sent to 629 Italian seafarers and we conducted analysis on a valid sample of 239 respondents.
- We added the following information in the text to better explain the difficulty in getting the actual numbers of the Italian seafarers: “determining the exact number of seafarers is Italy is challenging due to the lack of a public register. A report from Confitarma (2019) suggested an estimate of 46,350 seafarers in Italy (Duci et al., 2019).”.

# Reviewer report: Reviewer response for version 1

**DOI:** 10.5256/f1000research.174890.r358867

**Published:** 2025-01-21

**Authors:** Vairavan C

**Affiliations:** 1 AMET Deemed to be University, Chennai, Tamil Nadu, India

The article examines burnout and health impairment among Italian seafarers using the Job Demands-Resources (JD-R) model. This topic is highly relevant given the unique challenges faced by maritime professionals. The study offers significant contributions by focusing on the underexplored demographic of Italian seafarers and employing a structural equation model to analyze the mediating role of burnout.

 However, the manuscript has several areas that require improvement to ensure clarity, consistency, and alignment with the stated objectives. While the findings are meaningful, the presentation of data, methodology, and interpretations can be enhanced to increase the overall impact and reproducibility of the research.

**Specific Comments:**

**Abstract**

 The abstract effectively introduces the study but contains inconsistencies in reporting sample sizes. It mentions 239 respondents, yet the methods section refers to an initial sample of 629. This discrepancy must be clarified to maintain transparency and ensure the validity of the reported findings. Additionally, the abstract lacks key statistical highlights, such as path coefficients or confidence intervals, which would provide a more robust summary of results.

**Introduction**

 The introduction contextualizes the study well, emphasizing the distinct work conditions of seafarers and the relevance of the JD-R model. However, the transition from general observations to the theoretical framework feels abrupt. A more explicit linkage between the challenges of maritime work and the JD-R framework is needed to strengthen coherence.

**Literature Review**

 The review comprehensively covers prior studies on seafarers' mental health and the JD-R model. However, it does not critically evaluate gaps in existing research. For instance, while the review mentions studies on burnout and fatigue, it does not explain how the study addresses these gaps in the context of Italian seafarers. Including this analysis would enhance the rationale for the study.

**Methods**

 The methodology is adequately detailed but raises concerns regarding sampling and potential biases. The description of how participants were recruited—"through a dedicated website and union support"—requires elaboration. Were participants self-selected, or was random sampling used? This distinction affects the generalizability of the findings. Additionally, the use of a cross-sectional design limits causal interpretations, a limitation that is only briefly acknowledged in the discussion.

**Results**

 The results section is well-organized and presents the data effectively using tables and figures. However, these findings would benefit from additional interpretation within the text. For example, while the structural equation model results are statistically robust, the implications of specific fit indices and path coefficients are not thoroughly explained. Furthermore, some terminology, such as "C.I.," may be unfamiliar to non-specialist readers and should be briefly clarified.

**Discussion**

 The discussion highlights key findings, particularly the mediating role of burnout between job demands/resources and health impairments. While these findings are significant, their practical applications are underexplored. For example, the authors could elaborate on how transformational leadership training or enhanced social support systems could be implemented aboard ships to mitigate burnout and health impairments.

 Additionally, the discussion could benefit from a more explicit comparison with studies in other occupational contexts. The current text mentions incongruence with general worker populations but does not provide sufficient examples or interpretations to support this claim.

**Conclusion**

 The conclusion effectively summarizes the study’s key findings but is reiterative. It calls for longitudinal studies, which is appropriate, but the authors could also suggest immediate interventions based on their findings, such as policies to reduce cognitive load or improve crew relationships.

**Limitations**

 The limitations section is comprehensive but could further address the overlap between burnout and depression. This overlap is mentioned briefly, but its implications for the study's conclusions are not fully explored. Additionally, the absence of gender-based analysis due to the small number of female participants is a missed opportunity that could have been acknowledged more explicitly.

**References**

 The references are extensive and relevant. However, a few citations, such as those related to the JD-R model, are outdated. Incorporating more recent studies would strengthen the theoretical grounding.

Is the work clearly and accurately presented and does it cite the current literature?

Partly

If applicable, is the statistical analysis and its interpretation appropriate?

Yes

Are all the source data underlying the results available to ensure full reproducibility?

Yes

Is the study design appropriate and is the work technically sound?

Partly

Are the conclusions drawn adequately supported by the results?

Partly

Are sufficient details of methods and analysis provided to allow replication by others?

Yes

Reviewer Expertise:

Psychological well-being, burnout, and health impairment among maritime professionals.The application of the JD-R model to study job demands, resources, and mental health.Social structures, leadership styles, and challenges in the maritime industry. Wind-assisted propulsion technologies like Flettner Rotors and sails.Biodiesel production using waste cooking oil and coffee husks oil.

I confirm that I have read this submission and believe that I have an appropriate level of expertise to confirm that it is of an acceptable scientific standard, however I have significant reservations, as outlined above.

# Response

**Published:** 2025-01-27

**Authors:** Francesco Buscema

**Affiliations:** Psychology, University of Turin Department of Psychology, Turin, Piedmont, Italy

Thanks for the valuable revision, we addressed the issues according to your suggestions as follows:

- **Abstract **- Thank you for finding the mistake in the abstract, we clarified the total number of respondents by adding the following sentence in the abstract: “To investigate these phenomena, an online questionnaire was sent to 629 Italian seafarers and we conducted analysis on a valid sample of 239 respondents”. Moreover, we added path coefficients for the mediations in the results section of the abstract.
- **Introduction **- We added the following linkage between theory and context in the introduction as follow: “Stressors, such as prolonged isolation, demanding physical tasks and the psychological strain of working in a confined environment, are exacerbated by the limited availability of resources, such as social support and effective leadership, which are critical to mitigating the negative effects of these demands. Considering this, the JD-R model is particularly relevant in this context as it assumes that the balance between job demands and resources has a direct impact on employee wellbeing and stress levels.” […] “By applying this model to the maritime sector, we can better understand how specific job resources can alleviate the psychological and physical stress of seafarers and thus promote a healthier working environment.”
- **Literature Review** - Thank you, we made clear the literature gaps and how we addressed them with our study. We added the following sentences to the text: “Nevertheless, the number of studies dealing with these topics if still small and there are even fewer studies in the Italian context.”, “Our study will aim to deepen the knowledge of psychosocial risk factors and possible factors that mediate the well-being of seafarers.”, and “To the best of our knowledge, this study is the first to test the JD-R model on Italian seafarers, and thus enriches the international literature.”.
- **Methods** - We agreed with the reviewer that voluntary participation was not explicitly disclosed and therefore added the following sentence in the Methods section: “Respondents were recruited through snowball sampling and participation was voluntary.” Furthermore, we added in the conclusion that this kind of sampling impacts the representativity of the finding: “There are some limitations to this study. All data were collected using an online self-report questionnaire on a voluntary basis, which means that participant-reported effects may be biased, especially for sensitive topics such as health and psychological well-being”.
- **Results **- We added the full form of the acronym (Confidence Interval = C.I.) at the first occurrence. Regarding the fit indices, we already provided the thresholds in the text “CFI and TLI have values that are above the cut-off (0.90), while SRMR and RMSEA are acceptable as the values are below the cut-off (.08 and .05 respectively), indicating that the model has a good fit ( Hu & Bentler, 1999).”. Finally, regarding the path coefficient, we added the following sentences to better explain the results “Confirming the theoretical model of JD-R, job demands (work and cognitive load) were positively associated with burnout (estimate = 0.622, *p* < 0.001)., while job resources (social support and transformational leadership) were negatively associated with burnout (estimate = - 0.350, *p* < 0.001). Moreover, burnout was strongly and positively associated with health impairment (estimate = 0.713, *p* < 0.001).”
- **Discussion** - Thank you for the valuable suggestions, we took this opportunity to expand and deeper explain the discussion section adding indication to design leadership training and team-building activities. Moreover, we compared more explicitly our study population with other professions. See the last two paragraphs in the discussion.
- **Conclusion** - Thank you for recognising the importance of intervention in the maritime sector. We believe we have addressed this issue in the revised version by discussing leadership training and team-building activities to improve the well-being of seafarers and their social working environment.
- **Limitations - **We have added a better explanation of why it is still important to assess burnout in high-stress occupations, as stated in a recent review by Koutsimani et al. 2019. We have also added a reference to the actual gender distribution in the maritime sector.
- **References** - In reviewing the references, we have found that all citations appear to be current (such as Bakker et al., 2023), except for those that refer specifically to older models for comparison with the JD-R model.

# Reviewer report: Reviewer response for version 1

**DOI:** 10.5256/f1000research.174890.r358862

**Published:** 2025-01-17

**Authors:** Inga Bartuseviciene

**Affiliations:** 1 World Maritime University, Malmö, Sweden

The topic of the paper is relevant, because the research provides critical insights into the psychological well-being of Italian seafarers, highlighting the interplay between job demands, burnout, and health impairment, and underscores the urgent need for targeted interventions in the maritime industry to enhance seafarers' quality of life and work conditions. However, in my opinion, some of the issues related to the quality of the manuscript can be improved:

1. The information of the sample of the respondents should be provided consistently: in the Abstract – “online questionnaire was sent to 239 Italian seafarers“; in the methods section - “629 seafarers from all departments and ranks responded to the questionnaire” and “total valid sample for the present analysis was 239 seafarers”.
2. Providing more detailed socio-demographic characteristics of the respondents could be useful.
3. An explanation of the sampling method should be provided.
4. A critical analysis of why the used instruments are the most appropriate for the purpose of the research could be useful.
5. This statement is doubtful: “there is no information on the actual number of seafarers in Italy”, usually maritime administration of the country has information about the number of issued certificates. This might be useful to provide insight about the generalisability of the outcomes at least for the population of seafarers in Italy.

Is the work clearly and accurately presented and does it cite the current literature?

Yes

If applicable, is the statistical analysis and its interpretation appropriate?

Yes

Are all the source data underlying the results available to ensure full reproducibility?

Partly

Is the study design appropriate and is the work technically sound?

Partly

Are the conclusions drawn adequately supported by the results?

Yes

Are sufficient details of methods and analysis provided to allow replication by others?

Yes

Reviewer Expertise:

Maritime Education and training, well-being and resilience

I confirm that I have read this submission and believe that I have an appropriate level of expertise to confirm that it is of an acceptable scientific standard, however I have significant reservations, as outlined above.

## References

[1] : Through restful waters and deep commotion: A study on burnout and health impairment of Italian seafarers from the JD-R model perspective. *F1000Research* .2025;14: 10.12688/f1000research.159198.1 10.12688/f1000research.159198.1 PMC1175161239844846

# Response

**Published:** 2025-01-27

**Authors:** Francesco Buscema

**Affiliations:** Psychology, University of Turin Department of Psychology, Turin, Piedmont, Italy

Thanks for the valuable revision, we addressed the issues according to your suggestions as follows:

1. Thank you for finding the mistake in the abstract, we clarified the total number of respondents adding the following sentence in the abstract: “To investigate these phenomena, an online questionnaire was sent to 629 Italian seafarers, and we conducted analysis on a valid sample of 239 respondents”
2. We added two further information about the sample in the methods section: “Concerning the marital status of our sample, 47.7% were married, 25.1% were engaged, 20.5% were single, while the rest were divorced or widowed.” […] “Furthermore, 64.9% of the sample responded to the survey while on board.”
3. We added the following sentence in the section regarding data collection “Respondents were recruited through snowball sampling and participation was voluntary.” Furthermore, we added in the conclusion that this kind of sampling impact the representativity of findings.
4. Regarding workload and physical workload, we opted for the Italian adaptation of the Psychological Workload and Physical Workload subscales of the Job Content Questionnaire (JCQ), which is one of the standards in research following the JD-R model (Zappalà et al., 2008; Forcella et al., 2012; Ramaci, 2024). To clarify this, we have added the following sentence in the manuscript: “which is one of the most common instrument for assessing workload”. We also opted for the scales to assessing job resources based on the study by Yuen et al. (2020) mentioned in the manuscript. The measurement tool to assess burnout was selected based on a literature review in the maritime sector, with the most commonly used tool chosen to allow easy comparison with other studies. The measurement tools to assess health impairment were selected based on the study by Wan et al. (2023), adapting them to the Italian context.
5. Thank you for the valuable suggestion, we added the following sentence in the text: “determining the exact number of seafarers is Italy is challenging due to the lack of a public register. A report from Confitarma (2019) suggested an estimate of 46,350 seafarers in Italy (Duci et al., 2019).”
